# Oncological outcomes of neoadjuvant chemotherapy in patients with resectable synchronous colorectal liver metastasis: A result from a propensity score matching study

**DOI:** 10.3389/fonc.2022.951540

**Published:** 2022-10-18

**Authors:** Yu-Juan Jiang, Si-Cheng Zhou, Jing-Hua Chen, Jian-Wei Liang

**Affiliations:** ^1^ Department of Colorectal Surgery, National Cancer Center/National Clinical Research Center for Cancer/Cancer Hospital, Chinese Academy of Medical Sciences and Peking Union Medical College, Beijing, China; ^2^ Department of Hepatobiliary Surgery, National Cancer Center/National Clinical Research Center for Cancer/Cancer Hospital, Chinese Academy of Medical Sciences and Peking Union Medical College, Beijing, China

**Keywords:** colorectal liver metastasis, neoadjuvant chemotherapy, surgical resection, prognosis factors, propensity score matching

## Abstract

**Background:**

The efficacy and safety of neoadjuvant chemotherapy (NAC) in treating resectable synchronous colorectal liver metastases (CRLM) remain controversial.

**Methods:**

Data from CRLM patients who underwent simultaneous liver resection between January 2015 and December 2019 were collected from the Surveillance, Epidemiology, and End Results (SEER) database (SEER cohort, n=305) and a single Chinese Cancer Center (NCC cohort, n=268). Using a 1:2 ratio of propensity score matching (PSM), the prognostic impact of NAC for patients who underwent NAC before surgical treatment and patients who underwent surgical treatment alone was evaluated.

**Results:**

After PSM, there was no significant difference in overall survival (OS) between patients receiving NAC prior to CRLM resection and those undergoing surgery only, in both the NCC and SEER cohorts (each *P* > 0.05). Age was an independent predictor of OS only in the SEER cohort (*P* = 0.040), while the pN stage was an independent predictor for OS only in the NCC cohort (*P* = 0.002). Furthermore, Disease-free survival (DFS) was comparable between the two groups in the NCC cohort. In a subgroup analysis, the DFS and OS in the NAC- group were significantly worse than those in the NAC+ group for patients with more than two liver metastases in the NCC cohort (*P* < 0.05 for both).

**Conclusion:**

NAC did not have a significant prognostic impact in patients with resectable synchronous CRLM. However, patients with more than two liver metastases could be good candidates for receiving NAC.

## Introduction

Colorectal cancer (CRC) is the third most prevalent diagnosed malignant tumor worldwide and was the second most common cause of cancer death in 2020 (1). It is estimated that CRC comprises 10% (0.4 million) of new cancer cases and causes 8% (0.2 million) of cancer-related deaths in China (2). The liver is the most common organ of colorectal metastases, with approximately 25% of patients presenting with synchronous CRC with liver metastases (CRLM) at the time of diagnosis (3). Patients with untreated CRLM have a poor median survival of 4.5 months (4). Surgical resection of liver metastases is the only curative treatment for patients with CRLM, with a reported 5-year survival of 40%-51% (5, 6). However, postoperative recurrence of liver metastasis after hepatectomy is still widespread (50%-75%), especially in the remnant liver, and studies have reported a correlation between recurrence and inferior survival outcomes (7, 8). Due to advances in chemotherapy over the last decade, liver metastases that would be previously classified as unresectable may be reconsidered as resectable after neoadjuvant chemotherapy (NAC). Consequently, the outcome of CRLM has improved (9, 10). However, the evidence for NAC efficacy for patients with operable metastases is less obvious and has been a source of controversy for many years (11, 12).

The EORTC 40983 trial, which is the only randomized controlled trial of perioperative chemotherapy for patients with resectable CRLM, randomly assigned 364 patients to perioperative FOLFOX4 (5-fluorouracil/oxaliplatin) or surgery alone and showed some improvement in 3-year progression-free survival (PFS) in patients with perioperative NAC (13). However, the subsequent long-term findings revealed that the differences in 3- or 5-year overall survival (OS) rates between the two groups were not significant (14). Given that the EORTC 40983 trial has shown promising short-term outcomes, the oncology community has incorporated this practice worldwide (7). However, the updated National Comprehensive Cancer Network (NCCN) guidelines suggest both surgery alone and NAC for patients with resectable CRLM; there is no definite treatment guideline for CRLM. In recent years, several researchers have found that NAC offers no substantial survival advantage for patients with resectable CRLM; at the same time, NAC has also attracted extensive attention for its potential for chemotherapy-associated liver injury (15). As a result, the efficacy and safety of NAC for patients with resectable synchronous CRLM are still up for debate. This study aimed at comparing the effectiveness of NAC followed by radical resection versus surgical resection alone for resectable synchronous CRLM patients.

## Materials and methods

### Patient information

We reviewed consecutive patients with synchronous colorectal liver metastases who underwent simultaneous surgical resection of primary and liver metastatic lesions between January 2015 and December 2019 from the Surveillance, Epidemiology, and End Results (SEER) database. Patients were selected using the SEER*Stat software (version 8.3.8) from the database of ‘Incidence-SEER 18 Regs Custom Data (with additional treatment fields), May 2022 Sub (1975-2019 varying)’. According to ‘Histologic Type ICD-O-3’, the following pathological types were included in this study: adenocarcinoma (8010, 8020–8022, 8140–8141, 8144–8145, 8210–8211, 8220–8221, 8230–8231, 8260–8263), mucinous adenocarcinoma (8472, 8473, 8480, 8481) and signet ring cell carcinoma (8490). All staging data were pathologically validated and recorded using the AJCC TNM staging system, 7th edition. The inclusion criteria were as follows: (1) pathologically diagnosed synchronous CRLM between 2015-2019 and (2) underwent simultaneous surgical resection of primary and metastatic lesions; (3) patients aged 20 to 85. The exclusion criteria were as follows: (1) patients with extrahepatic metastasis; (2) patients with incomplete clinic pathological information (pT stage, pN stage, histologic type, chemotherapy record); (3) patients with incomplete follows; (4) patients with R2 (macroscopic residual disease) resection, or radio frequency ablation (PFA). The final SEER total cohort included in the analysis has 305 cases.

**Figure 1:**
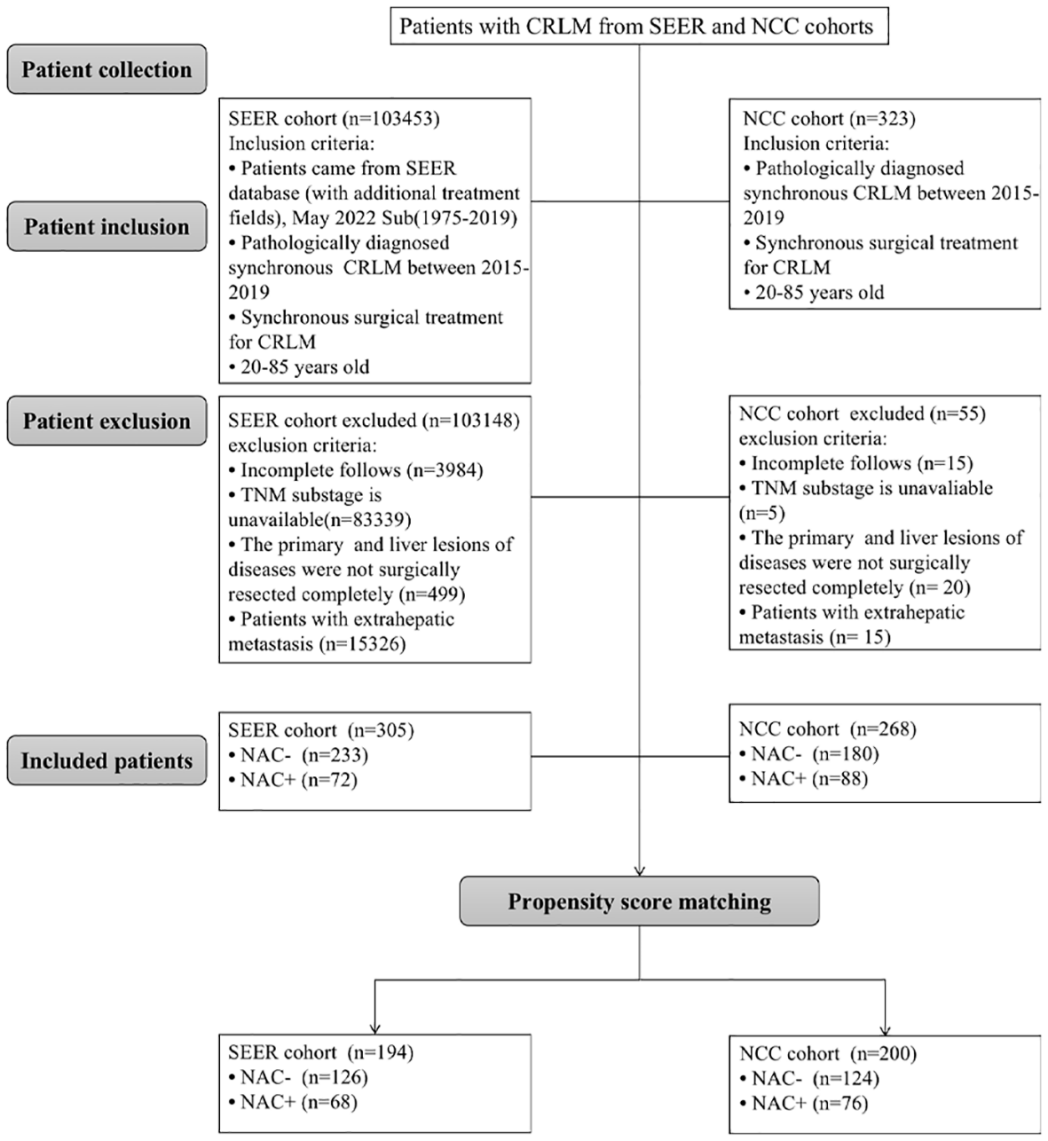
Flow diagram of the study.

CRLM patients who underwent surgery for the primary lesions and liver metastases simultaneously at the National Cancer Center (NCC) between January 2015 and December 2019 were collected using the same exclusion and inclusion criteria used for searching the SEER database. Patients who received less than three cycles of neoadjuvant chemotherapy were also excluded from the study. Finally, 268 patients met the inclusion criteria and were included in the study, and each patient provided informed consent. Multidisciplinary team meetings involving medical and surgical oncologists determined treatment strategies for each patient based on their wishes. The National Cancer Center’s Institute Research Medical Ethics Committee approved this study (NCC 2017-YZ-026, 17 October 2017). The screening process is shown in **Figure 1**.

### Chemotherapy regimen

Physicians recommended neoadjuvant chemotherapy based on the patient’s condition, clinical manifestations, laboratory tests, and imaging findings. Subsequently, patients chose whether to receive NAC after being told that the efficacy of NAC for CRLM was debated. Finally, 88 patients received NAC with regimens of either FOLFIRI (leucovorin, fluorouracil, and irinotecan); or FOLFOX (leucovorin, 5-fluorouracil, and oxaliplatin); or XELOX (capecitabine and oxaliplatin), with or without targeted agents (bevacizumab, cetuximab or panitumumab) in the NCC cohort. The surgery was performed 4 to 6 weeks following the final round of treatment. The number of chemotherapy cycles varied according to the physicians’ discretion. In our center, adjuvant chemotherapy was routinely recommended for CRLM after discharge from the hospital. For patients who underwent preoperative treatment, at least six months of perioperative chemotherapy was performed regardless of the pathological stage. The chemotherapy regimens were presented in **Supplementary Table 1**.

### Definitions

CRLM was defined as synchronous when metastases were discovered during pre-therapeutic staging or surgery for the primary tumor. In the NCC cohort, resectable CRLM was defined by the following characteristics: (1) tumors that could be physically resected, leaving intact at least 30% of the remaining liver volume; (2) the H1‐factor scale according to the Japanese Classification of Colorectal Carcinoma from the Japanese Society for Cancer of the Colon and Rectum (one to four metastatic lesions with a maximum diameter of 5 cm or less) (16). For resectable CRLM in the SEER database, the following options were used: RXSumm-SurgPrim (Surgery of Primary Site), RX Summ–Surg Oth Reg/Dis (Surgical Procedure of Other Site), SEER Combined Mets at DX-liver (Liver is an involved metastatic site at diagnosis) (17). The interval between operation and observation of disease progression or death was identified as disease-free survival (DFS). The dates of surgery and the latest known follow-up or death were used to calculate OS. The primary endpoints of this study are OS. Secondary endpoints included DFS and perioperative outcomes.

### Surgical procedure

Before surgery, all patients were diagnosed with histologically established CRC based on a colonoscopic sample. Computed tomography (CT) and dynamic magnetic resonance imaging (MRI) of the liver were used to assess the primary tumor and metastatic lesions. Positron emission tomography (PET) was performed on a limited number of individuals as needed. An experienced team of surgeons from the colorectal and hepatobiliary departments performed all of the operations under general anesthesia. Hepatic ultrasonography was not commonly used intraoperatively because MRI was used to assess liver metastases before surgery. Standard CRC resections were conducted strictly following the cancer surgery guidelines for both open and laparoscopic procedures. Concerning liver resection, if possible, partial resection of the liver was chosen; if not, segment resection or lobectomy were selected to preserve liver function. In the event of bleeding, the Pringle technique was used selectively. All patients underwent potentially curative CRLM resection, defined as complete tumor excision with a negative macroscopic margin.

### Follow−up

After surgery, patients were screened every three months for the first two years, every six months for the second to the fifth years, and once a year after that. Tumor markers, endoscopy with or without biopsy, and chest and thoracoabdominal CT were evaluated. Progressive soft-tissue growth and hypermetabolic lesions revealed by CT were considered tumor recurrence. The follow-up data were reviewed by December 1, 2021.

### Statistics

**Figure 2:**
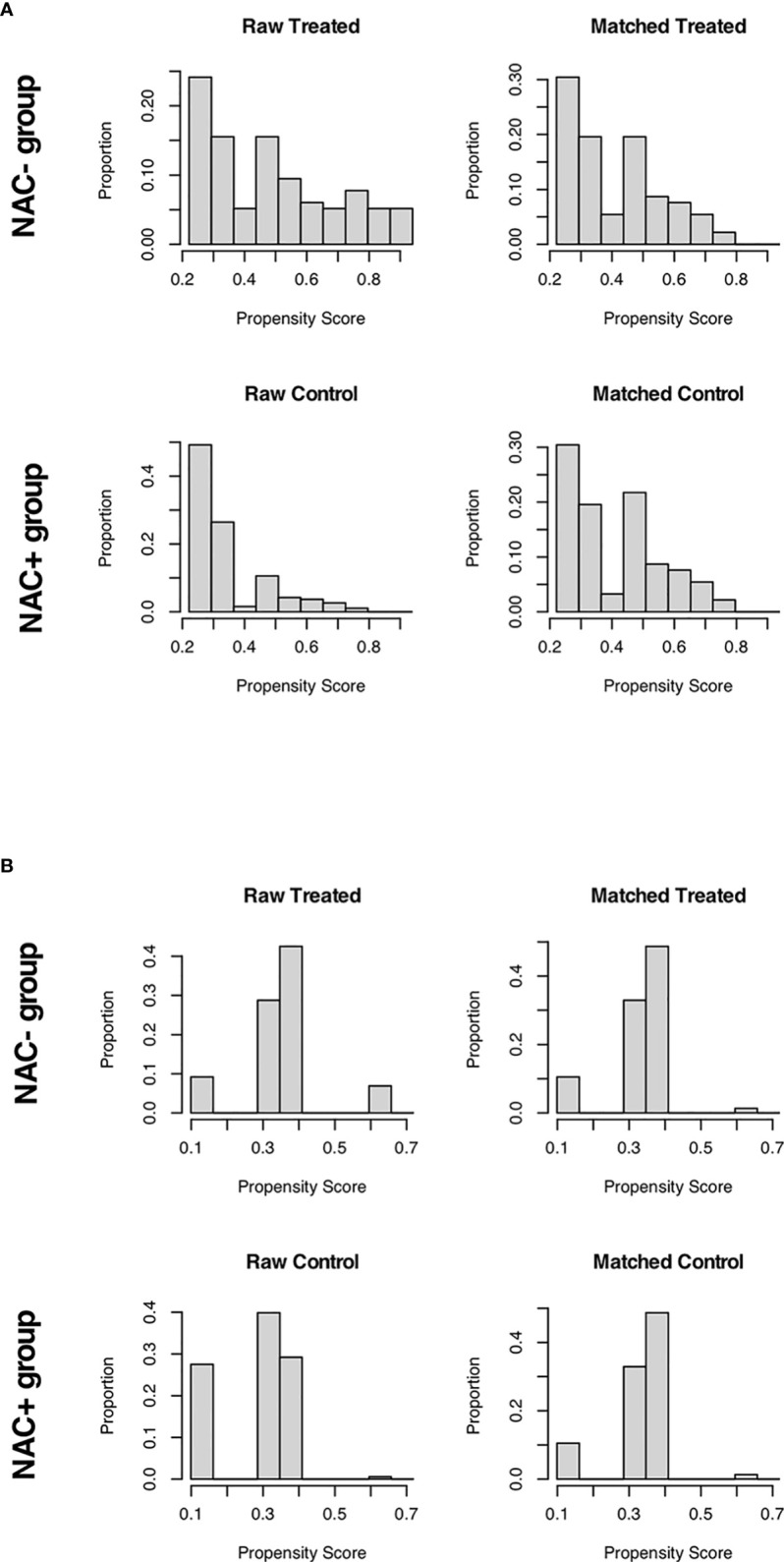
Distribution of propensity scores across the two groups: SEER cohort **(A)**; NCC cohort **(B)**.

For patients receiving NAC and not, a 1:2 propensity score matching (PSM) analysis was conducted to balance the imbalanced covariates (*P* < 0.05) between the two groups. In the NCC cohort, the site of primary disease and pT stage were used as matching criteria; while the site of primary disease, pT stage, and pN stage were matching criteria in the SEER cohort (each *P* < 0.05). The nearest neighbor matching approach was used with a caliper width of 0.20 to achieve a 1:2 ratio match between groups (**Figure 2**). Continuous variables were expressed as median (range) values and were compared using the t-test. The chi-square test and Fisher’s exact test were used to compare categorical variables expressed as numbers with percentages. Survival rates were estimated using the Kaplan–Meier method and were compared using the log-rank test. The Cox proportional hazards model was used for univariate and multivariate analyses. Multivariate analysis was used to examine the significant variables (*P* < 0.1) in the univariate analysis. *P* < 0.05 was considered to denote statistical significance for all analyses. All analyses were performed using the R software, version 3.5.1 (http://www.r-project.org/).

## Results

### Baseline characteristics

Baseline characteristics of CRC patients with synchronous liver metastasis in the SEER cohort and NCC cohort were summarized in **Supplementary Table 2**. In the NCC cohort, 72 patients (26.9%) had liver surgery first, while 196 (73.1%) had colorectal surgery first. Of the 268 NCC cohort patients, 88 underwent NAC before primary cancer and metastatic lesions excision, and the demographics and tumor characteristics for these patients were presented in **Table 1**. The results showed that before PSM analysis, patients undergoing NAC were more common in colon cancer (*P* < 0.001) and pT1/T2 stage (*P* = 0.003). However, after a 1:2 matched PSM analysis, there was no statistically significant variation in the distribution of baseline characteristics between the NAC+ group (n = 76) and NAC- group (n = 124). The baseline characteristics of CRLM patients in the SEER database were summarized in **Table 2**, revealing that the baseline characteristics of the two groups did not match. Based on the 1:2 matched PSM analysis, 194 CRLM patients treated with NAC first (n = 68) or surgical resection first (n = 126) were included in the survival analysis. The baseline characteristics of the two groups were nearly identical.

**Table 1:** Baseline clinical characteristics of NCC cohort.

Characteristics	Before PSM			After PSM		
	Patients without NAC(n = 180)	Patients with NAC(n = 88)	*P-value*	Patients without NAC(n = 124)	Patients with NAC(n = 76)	*P-value*
**Age (years)**			0.630			0.871
Mean (SD)	58.3 (9.91)	57.6 (10.1)		57.8 (9.88)	57.6 (10.2)	
Median [Min, Max]	60.0 [21.0, 78.0]	58.0 [30.0, 76.0]		60.0 [21.0, 78.0]	58.5 [30.0, 75.0]	
**Sex (%)**			0.479			1.000
Male	107 (59.4%)	57 (64.8%)		80 (64.5%)	49 (64.5%)	
Female	73 (40.6%)	31 (35.2%)		44 (35.5%)	27 (35.5%)	
**ASA scores (%)**			0.671			0.662
I	8 (4.4%)	2 (2.3%)		6 (4.8%)	2 (2.6%)	
II	155 (86.1%)	78 (88.6%)		106 (85.5%)	68 (89.5%)	
III	17 (9.4%)	8 (9.1%)		12 (9.7%)	6 (7.9%)	
**Number of liver metastases (%)**			0.243			0.335
<3	119 (66.1%)	51 (58.0%)		63 (50.8%)	45 (59.2%)	
≥3	61 (33.9%)	37 (42.0%)		61 (49.1%)	31 (40.7%)	
**Size of liver metastases (%)**			0.282			0.168
<3cm	118 (65.6%)	51 (58.0%)		61 (49.2%)	31 (40.8%)	
≥3cm	62 (34.4%)	37 (42.0%)		63 (50.8%)	45 (59.2%)	
**Comorbidity (%)**			0.884			0.838
Absent	97 (53.9%)	49 (55.7%)		67 (54.0%)	43 (56.6%)	
Present	83 (46.1%)	39 (44.3%)		57 (46.0%)	33 (43.4%)	
**Site of primary disease (%)**			**<0.001**			0.529
Rectum	56 (31.1%)	9 (10.2%)		20 (16.1%)	9 (11.8%)	
Colon	124 (68.9%)	79 (89.8%)		104 (83.9%)	67 (88.2%)	
**Histologic grade (%)**			0.578			0.968
Poor/Mucinous/signet	40 (22.2%)	23 (26.1%)		31 (25.0%)	20 (26.3%)	
Moderate	140 (77.8%)	65 (73.9%)		93 (75.0%)	56 (73.7%)	
**pN stage (%)**			0.364			0.896
N0	54 (30.0%)	32 (36.4%)		40 (32.3%)	26 (34.2%)	
N+	126 (70.0%)	56 (63.6%)		84 (67.7%)	50 (65.8%)	
**pT stage (%)**			**0.003**			0.983
T1/T2	13 (7.2%)	18 (20.5%)		10 (8.1%)	7 (9.2%)	
T3/T4	167 (92.8%)	70 (79.5%)		114 (91.9%)	69 (90.8%)	
**Adjuvant chemotherapy (%)**			0.807			0.724
No	26 (14.4%)	11 (12.5%)		51 (41.1%)	34 (44.7%)	
Yes	154 (85.6%)	77 (87.5%)		73 (58.9%)	42 (55.3%)	

PSM, Propensity scoring matching; NAC, neoadjuvant chemotherapy; pT: pathologic T stage; pN, pathologic N stage. Statistically significant p-values are indicated in bold.

**Table 2:** Baseline clinical characteristics of SEER cohort.

Characteristics	Before PSM			After PSM		
	Patients without NAC(n = 233)	Patients with NAC(n = 72)	*P-value*	Patients without NAC(n = 126)	Patients with NAC(n = 68)	*P-value*
**Age (years)**			0.204			0.204
Mean (SD)	57.3 (11.4)	58.9 (8.18)		57.2 (11.5)	59.1 (8.37)	
Median [Min, Max]	55.0 [31.0, 81.0]	55.0 [40.0, 82.0]		55.0 [31.0, 80.0]	55.0 [40.0, 82.0]	
**Sex (%)**			1.000			0.722
Male	138 (59.2%)	42 (58.3%)		77 (61.1%)	39 (57.4%)	
Female	95 (40.8%)	30 (41.7%)		49 (38.9%)	29 (42.6%)	
**Race (%)**			0.615			0.520
White	177 (76.0%)	58 (80.6%)		96 (76.2%)	56 (82.4%)	
Other	27 (11.6%)	8 (11.1%)		17 (13.5%)	8 (11.8%)	
Black	29 (12.4%)	6 (8.3%)		13 (10.3%)	4 (5.9%)	
**Site of primary disease (%)**			**0.006**			0.961
Rectum	56 (24.0%)	30 (41.7%)		50 (39.7%)	28 (41.2%)	
Colon	177 (76.0%)	42 (58.3%)		76 (60.3%)	40 (58.8%)	
**Histologic grade (%)**			0.374			0.348
Poor/Mucinous/signet	210 (90.1%)	68 (94.4%)		112 (88.9%)	64 (94.1%)	
Moderate	23 (9.9%)	4 (5.6%)		14 (11.1%)	4 (5.9%)	
**pN stage (%)**			**0.005**			0.717
N0	47 (20.2%)	27 (37.5%)		38 (30.2%)	23 (33.8%)	
N+	186 (79.8%)	45 (62.5%)		88 (69.8%)	45 (66.2%)	
**pT stage (%)**			**0.001**			0.435
T1/T2	16 (6.9%)	15 (20.8%)		14 (11.1%)	11 (16.2%)	
T3/T4	217 (93.1%)	57 (79.2%)		112 (88.9%)	57 (83.8%)	
**Adjuvant chemotherapy (%)**			0.946			0.444
No	81 (34.8%)	26 (36.1%)		53 (42.1%)	24 (35.3%)	
Yes	152 (65.2%)	46 (63.9%)		73 (57.9%)	44 (64.7%)	

PSM, Propensity scoring matching; NAC, neoadjuvant chemotherapy; pT: pathologic T stage; pN, pathologic N stage. Statistically significant p-values are indicated in bold.

### Short-term recovery of NCC cohort

Perioperative outcomes of the NAC and surgical resection groups were shown in **Supplementary Table 3**. Before and after PSM, the two groups did not significantly differ in the type of surgery, surgical margin, postoperative complications, blood loss, exhaust time, surgical margin, and length of hospital stay (*P* > 0.05 for each parameter). Only operation time was significantly longer in the NAC group than in the surgical resection group (350 min vs. 278 min, *P* < 0.001).

### Oncologic outcomes in the SEER cohort

**Figure 3:**
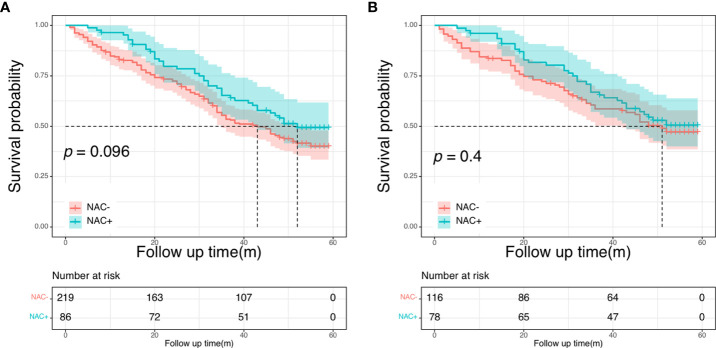
Overall survival curves in SEER cohort: in the entire cohort **(A)**; in the propensity score-matched cohort **(B)**.

Across the entire cohort, the 5-year OS rate was 49.3% for patients in the NAC+ group, compared with 40.0% for those in the NAC- group (*P* = 0.096) (**Figure 3A**). In the PSM cohort, the 5-year OS rate was 50.5% for patients in the NAC+ group, compared with 47.3% for those in the NAC- group (*P* = 0.400) (**Figure 3B**). In the entire cohort, the Cox multivariate analysis demonstrated that age (HR, 1.40; *P* = 0.037), histologic grade (HR, 0.56; *P* = 0.018), and site of primary disease (HR, 0.64; *P* = 0.011) were independent significant prognostic factors of OS (**Supplementary Figure 1A**) (**Table 3**). However, only age was an independent prognostic factor of OS in the PSM cohort (HR, 1.54; *P* = 0.040) (**Supplementary Figure 1B**) (**Table 3**).

**Table 3:** Prognostic factors for OS in CRLM patients in SEER cohort.

Characteristics	Univariate analyses		Multivariate analyses	
	*P-value*	HR (95% CI)	*P-value*	HR (95% CI)
***SEER cohort-before PSM***				
Age (≥60y vs <60y)	0.069	1.34 (0.98 - 1.84)	**0.037**	1.40 (1.02 - 1.93)
Sex (female vs male)	0.475	0.89 (0.66 - 1.22)		
Race (Black vs White)	0.775	1.08 (0.65 - 1.79)		
(Other vs White)	0.703	1.09 (1.65 - 1.79)		
pN stage (N+ vs N0)	**0.016**	1.63 (1.10 - 2.42)	0.064	1.47 (0.98 - 2.21)
pT stage (T3+T4 vs T1+T2)	**0.014**	2.23 (1.18 - 4.24)	0.056	1.89 (0.98 - 3.64)
Histologic grade (Poor/Mucinous/signet vs Moderate)	**0.004**	0.49 (0.30 - 0.80)	**0.018**	0.56 (0.34 - 0.90)
Site (Rectum vs Colon)	**0.009**	0.61 (0.43 - 0.88)	**0.011**	0.64 (0.43- 0.90)
Neoadjuvant Chemotherapy therapy (Yes vs No)	0.098	0.74 (0.52 - 1.06)	0.565	0.90 (0.63 - 1.29)
Adjuvant Chemotherapy therapy (Yes vs No)	0.948	0.99 (0.72 - 1.36)		
***SEER cohort-after PSM***	0.685	1.09 (0.73 - 1.63)		
Age (≥60y vs <60y)	**0.041**	1.53 (1.02 - 2.30)	**0.040**	1.54 (1.02 - 2.32)
Sex (female vs male)	0.119	0.72 (0.48 - 1.09)		
Race (Black vs White)	0.270	1.40 (0.77 - 2.52)		
(Other vs White)	0.808	1.09 (0.55 - 2.18)		
pN stage (N+ vs N0)	0.137	1.42 (0.89 - 2.26)		
pT stage (T3+T4 vs T1+T2)	0.070	1.96 (0.95 - 4.04)	0.086	1.90 (0.91 - 3.94)
Histologic grade (Poor/Mucinous/signet vs Moderate)	**0.032**	0.50 (0.27 - 0.94)	0.072	0.56 (0.30 - 1.05)
Site (Rectum vs Colon)	0.109	0.71 (0.46 - 1.08)		
Neoadjuvant Chemotherapy therapy (Yes vs No)	0.406	0.84 (0.55 - 1.27)		
Adjuvant Chemotherapy therapy (Yes vs No)	0.235	0.78 (0.52 - 1.17)		

PSM, Propensity scoring matching; pT: pathologic T stage; pN, pathologic N stage. Statistically significant p-values are indicated in bold.

### Oncologic outcomes in the NCC cohort

**Figure 4:**
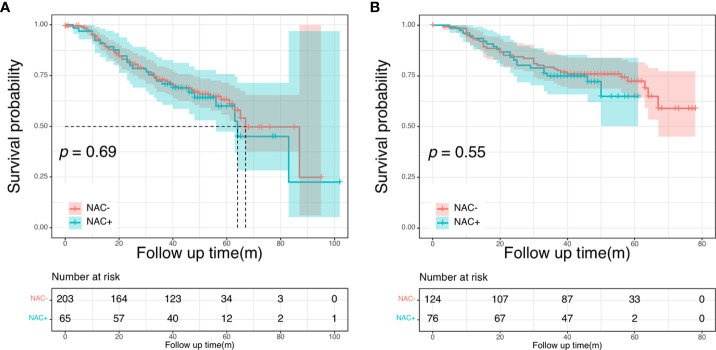
Overall survival curves in the NCC cohort: in the entire cohort **(A)**; in the propensity score-matched cohort **(B)**.

**Figure 5:**
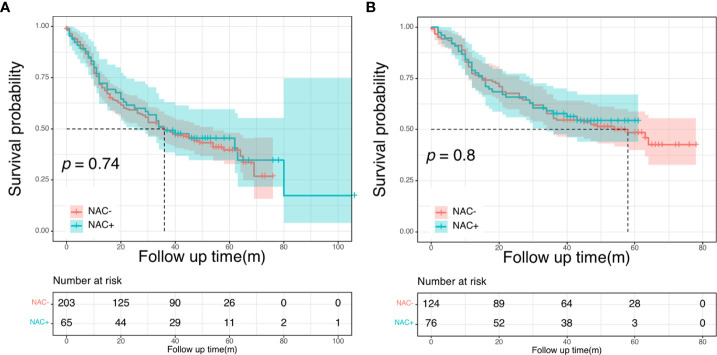
Disease-free survival curves in the NCC cohort: in the entire cohort **(A)**; in the propensity score-matched cohort **(B)**.

There were no differences in the 5-year OS rate between the NAC+ and NAC- before and after PSM (before PSM: 59.9% vs. 63.1%, P = 0.690; after PSM: 64.9% vs. 72.4%, *P* = 0.550) (**Figure 4**). The postoperative 5-year DFS rate was also not better in the NAC+ group than in the NAC- group, either before or after PSM (before PSM: 45.5% vs. 39.7%, *P* = 0.740; after PSM: 54.4% vs. 48.5%, *P* = 0.800) (**Figure 5**). Since the prognostic role of NAC was found to be not significant, peri- or post-operative events were examined. Multivariable analysis showed that age > 60 years (HR, 1.64; *P* = 0.017), pN+ stage (HR, 2.11; *P* = 0.003), rectal cancer (HR, 1.96; *P* = 0.002), number of liver metastases ≥ 3 (HR, 1.75; *P* = 0.021), and size of liver metastases ≥ 3 cm (HR, 1.73; *P* = 0.012) were independent prognostic predictors of OS (**Supplementary Figure 2A**) (**Table 4**). A 1:2 PSM analysis was conducted to balance covariates and avoid the selection bias of the retrospective study. IIn the PSM cohort, the pN stage was the only independent factor predicting worse OS outcomes (HR, 3.28; *P* = 0.002) (**Supplementary Figure 2B**) (**Table 4**).

**Table 4:** Prognostic factors for OS in CRLM patients in the NCC cohort.

Characteristics	Univariate analyses		Multivariate analyses	
	*P-value*	HR (95% CI)	*P-value*	HR (95% CI)
***NCC cohort-before PSM***				
Age (≥60y vs <60y)	**0.046**	1.51 (1.01 - 2.25)	**0.017**	1.64 (1.09 - 2.47)
Sex (female vs male)	0.356	0.82 (0.54 - 1.24)		
ASA (II vs I)	0.319	2.04 (0.50 - 8.32)		
(III vs I)	0.485	1.74 (0.37 - 8.18)		
pN stage (N+ vs N0)	**0.007**	1.98 (1.21 - 3.24)	**0.003**	2.11 (1.28 - 3.47)
pT stage (T3+T4 vs T1+T2)	0.436	1.31 (0.66 - 2.62)		
Histologic grade (Poor/Mucinous/signet vs Moderate)	0.228	1.33 (0.84 - 2.10)		
Size of liver metastases (≥3cm vs <3cm)	**0.011**	1.73 (1.13 - 2.64)	**0.012**	1.73 (1.13 - 2.66)
Site (Rectum vs Colon)	**<0.001**	2.13 (1.40 - 3.24)	**0.002**	1.96 (1.28 - 2.99)
Neoadjuvant Chemotherapy therapy (Yes vs No)	0.685	1.10 (0.70 -1.72)		
Adjuvant Chemotherapy therapy (Yes vs No)	0.960	0.96 (0.64 - 1.44)		
Comorbidity (Yes vs No)	0.685	1.09 (0.73 - 1.63)		
Complication (Yes vs No)	0.273	1.27 (0.83 - 1.96)		
Number of liver metastases (≥3 vs <3)	**0.039**	1.64 (1.03 - 2.61)	**0.021**	1.75 (1.09 - 2.80)
***NCC cohort-after PSM***				
Age (≥60y vs <60y)	0.127	1.51 (0.89 - 2.56)		
Sex (female vs male)	0.684	1.12 (0.64 - 1.77)		
ASA (II vs I)	0.315	2.77 (0.38 - 20.07)		
(III vs I)	0.376	2.64 (0.31 - 22.63)		
pN stage (N+ vs N0)	**0.002**	3.29 (1.56 - 6.97)	**0.002**	3.28 (1.55 - 6.95)
pT stage (T3+T4 vs T1+T2)	0.104	3.23 (0.79 - 13.3)		
Histologic grade (Poor/Mucinous/signet vs Moderate)	0.107	1.59 (0.90 - 2.80)		
Size of liver metastases (≥3cm vs <3cm)	0.211	0.71 (0.42 - 1.21)		
Site (Rectum vs Colon)	0.855	1.07 (0.51 - 2.27)		
Neoadjuvant Chemotherapy therapy (Yes vs No)	0.556	1.18 (0.68 -2.07)		
Adjuvant Chemotherapy therapy (Yes vs No)	0.374	0.68(0.29 - 1.59)		
Comorbidity (Yes vs No)	0.872	1.04 (0.61 - 1.77)		
Complication (Yes vs No)	0.084	1.62 (0.94 - 2.82)	0.088	1.62 (0.93 - 2.80)
Number of liver metastases (≥3 vs <3)	0.459	1.27 (0.67 - 2.42)		

PSM, Propensity scoring matching; pT: pathologic T stage; pN, pathologic N stage. Statistically significant p-values are indicated in bold.

Cox univariate and multivariate analyses were also performed to find the most significant prognostic factors of DFS. In the entire NCC cohort, pN stage (HR, 1.95; *P* < 0.001), number of liver metastases (HR, 1.86; *P* = 0.001), size of liver metastases (HR, 1.99, *P* < 0.001), and histologic grade (HR, 1.68, *P* = 0.007) were found to be independent prognostic factors for DFS in multivariate analysis (**Supplementary Figure 3A**) (**Supplementary Table 4**). After PSM, the pN stage (HR, 2.14, *P* = 0.002), histologic grade (HR, 2.14, *P* < 0.001) and size of liver metastases (HR, 0.53, *P* = 0.003) were identified as independent prognostic factors for worse DFS (**Supplementary Figure 3B**) (**Supplementary Table 4**).

### Subgroup analysis

**Figure 6:**
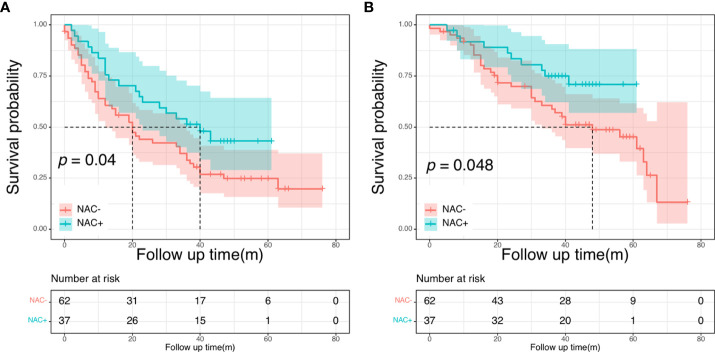
Disease-free survival curves **(A)** and overall survival **(B)** curves in patients with more than two liver metastases.

Next, we conducted the subgroup analysis based on the number of metastases further evaluate the prognostic impact of NAC in the NCC cohort. The clinical and pathological characteristics were presented in **Supplementary Table 5** before PSM. There were no significant differences between patients with <3 metastases and patients with ≥ 3 metastases. In patients with less than three liver metastases, both DFS and OS intervals showed no significant differences between the NAC+ and the NAC- groups (*P* = 0.273 and *P* = 0.420, respectively). Subsequently, we focused on a subgroup cohort that exclusively enrolled patients with more than two liver metastases. Overall, 99 patients had more than two liver metastases, including 62 patients in the NAC- group and 37 in the NAC+ group. The respective 5-year DFS rates were 43.1% for the NAC+ group and 24.8% for the NAC- group. The median DFS was significantly shorter for the NAC- group (20 months) than for the NAC+ group (40 months, *P* = 0.040; **Figure 6A**). The respective 5-year OS rates were 70.9% for the NAC+ group and 45.2% for the NAC- group. Overall survival was significantly shorter in the NAC- group than in the NAC+ group (*P* = 0.048, **Figure 6B**).

## Discussion

In the present study, using multi-center data and propensity score matching analysis, we evaluated the oncologic outcomes of treatments with and without NAC for patients with resectable synchronous CRLM. Our results revealed that NAC has little prognostic impact on patients with resectable synchronous CRLM. These findings imply that upfront synchronous surgery could be considered for individuals with resectable synchronous CRLM. However, NAC would provide a survival benefit in patients bearing more than two liver metastases.

The synchronous resection has several advantages, including a single approach and general anesthesia, and a shorter length of hospital stay (18). Furthermore, the postoperative outcomes of simultaneous resection appear to be similar to staged approaches (19). Thus, simultaneous resection of the primary CRC and the liver metastases is a broadly accepted approach. Several previous reports have presented varying degrees of evidence for the impact of NAC in patients with resectable CRLM, however, there is no definitive therapy recommendation for CRLM because of the limited randomized data comparing the outcome of treatments including NAC or not after surgical resection. The phase III EORTC 40,983 trial, which comprises the landmark prospective randomized controlled trial in the field, revealed that NAC increased PFS compared with surgery alone in patients with resectable CRLM (13), and based on this, both NCCN and European Society for Medical Oncology endorsed perioperative NAC for CRLM (20, 21). However, Nordlinger et al. continued their analysis to evaluate long-term overall survival in patients who had perioperative chemotherapy or received surgery alone and found no survival benefit of NAC at the 5-year follow-up (*P* = 0.303) (14). Therefore, it is still debatable if NAC can improve the overall treatment outcome in CRLM patients.

According to a review of the literature and a meta-analysis, neoadjuvant treatment did not offer a significant benefit when the liver disease was upfront resectable (22, 23). Univariable and multivariable analyses from several studies also found no link between NAC and survival outcomes (24, 25). However, these were all single-center investigations. Additionally, NAC is usually reserved for patients with advanced malignancies. As a result, variable background characteristics may result in selection bias and lead to incorrect results. In the current study, despite being retrospective, the participants were drawn from two separate cohorts receiving different treatment regimens for resectable CRLM. Additionally, we performed PSM to guarantee that the background data was consistent between patients with NAC and those who did not receive NAC. Our long-term prognostic results revealed that NAC did not improve potential treatment benefits, even after the background characteristics of the two groups were matched *via* PSM. Our findings are consistent with recent reviews and meta-analyses showing that NAC offered no survival benefit for patients with resectable tumors. Furthermore, we determined that the operating time was substantially longer in patients who underwent NAC (*P* < 0.001). As previously stated, NAC causes fibrosis in sphincters and damage to the neurovascular bundle of capillaries, which might increase the difficulty of surgery, slowing the procedure (26).

In the SEER cohort, age was an independent prognostic risk factor. Overall, there has been some debate about whether age can be a significant predictive factor in CRC. Various studies have observed a poorer survival for younger patients with CRC, which could be related to delayed diagnosis with advanced disease. In contrast, others assume it is attributable to young adults possessing more histologically advanced cancer (27, 28). Yet, other authors have suggested that the prognosis of younger patients with surgically treated CRC is similar to or better than that of the elderly. This tendency could be explained by the fact that younger patients are more likely to have an excellent general health status and endure more rigorous treatments. Our study found that older patients have poorer OS than young patients (*P* = 0.040). However, because of the small number of young patients with severe conditions in our dataset, these findings should be interpreted with caution. More extensive studies are needed to further understand age-related differences in CRLM prognosis.

The number of colorectal liver metastases is used by clinicians (along with the location of colorectal liver metastasis) to determine oncological and technical resectability (29). The number of liver metastases has also been incorporated into many prognostic rating systems that predict survival outcomes in patients with colorectal liver metastases (30). For example, Rees et al. reported that more than three hepatic metastases was an independent predictor of poor survival for metastatic colorectal cancer (31). However, there is currently no report on the relationship between the effectiveness of neoadjuvant therapy and the number of liver metastases. In our study, subgroup analysis showed no association between NAC and survival benefits in patients with less than three hepatic metastases. However, the DFS and OS were significantly better in the NAC+ group than in the NAC- group for patients with more than two liver metastases. There are no ideal indication criteria for NAC in patients with CRLM. Our findings imply that stratifying patient risk profiles (based on the number of liver metastases) is critical for maximizing the advantages of NAC in patients with resectable CRLM. Multi-institutional trials are required to optimize CRLM patient selection for NAC.

Some shortcomings of the present study need to be acknowledged. First, this was a retrospective analysis. Second, we did not investigate the data of patients who received NAC because their tumors were initially judged to be potentially resectable, but did not undergo surgery because the tumors were later found to be unresectable. Third, the two cohorts differed in terms of demographic characteristics. Finally, due to missing data in the SEER database, we were unable to investigate some other important factors, such as the detail of liver metastases, surgical outcome, chemotherapy regimen and dose, and DFS records. Nevertheless, even though it is based on retrospective data, this study is significant because a propensity score matching technique was used to assess many multi-center patients. As a result, we consider that this study provides a high level of evidence on the clinical outcomes of patients with resectable CRLM.

## Conclusion

In conclusion, we found that NAC did not significantly improve oncologic outcomes in patients with resectable synchronous CRLM, suggesting that upfront surgery without NAC was a viable choice. However, patients with more than two liver metastases could be good candidates for indication of NAC.

## Data availability statement

The original contributions presented in the study are included in the article/**Supplementary Material**. Further inquiries can be directed to the corresponding author.

## Ethics statement

The studies involving human participants were reviewed and approved by The National Cancer Center’s Institute Research Medical Ethics Committee approved this study (NCC 2017-YZ-026, 17 October 2017). The patients/participants provided their written informed consent to participate in this study.

## Author contributions

(I) conception and design: Y-JJ, S-CZ. (II) administrative support: J-WL. (III) provision of study materials or patients: Y-JJ and J-WL. (IV) collection and assembly of data: S-CZ. (V) data analysis and interpretation: S-CZ and J-HC. All authors contributed to the article and approved the submitted version.

## Acknowledgments

We would like to thank TopEdit (www.topeditsci.com) for its linguistic assistance during the preparation of this manuscript.

## Conflict of interest

The authors declare that the research was conducted in the absence of any commercial or financial relationships that could be construed as a potential conflict of interest.

## Publisher’s note

All claims expressed in this article are solely those of the authors and do not necessarily represent those of their affiliated organizations, or those of the publisher, the editors and the reviewers. Any product that may be evaluated in this article, or claim that may be made by its manufacturer, is not guaranteed or endorsed by the publisher.
