# Supplementary material for: Oncological outcomes of neoadjuvant chemotherapy in patients with resectable synchronous colorectal liver metastasis: A result from a propensity score matching study
Source: Front Oncol. 2022 Oct 18;12:951540. doi: 10.3389/fonc.2022.951540 (PMC9623041; doi:10.3389/fonc.2022.951540)
Supplement: Supplementary file 1 [file DataSheet_1.docx]

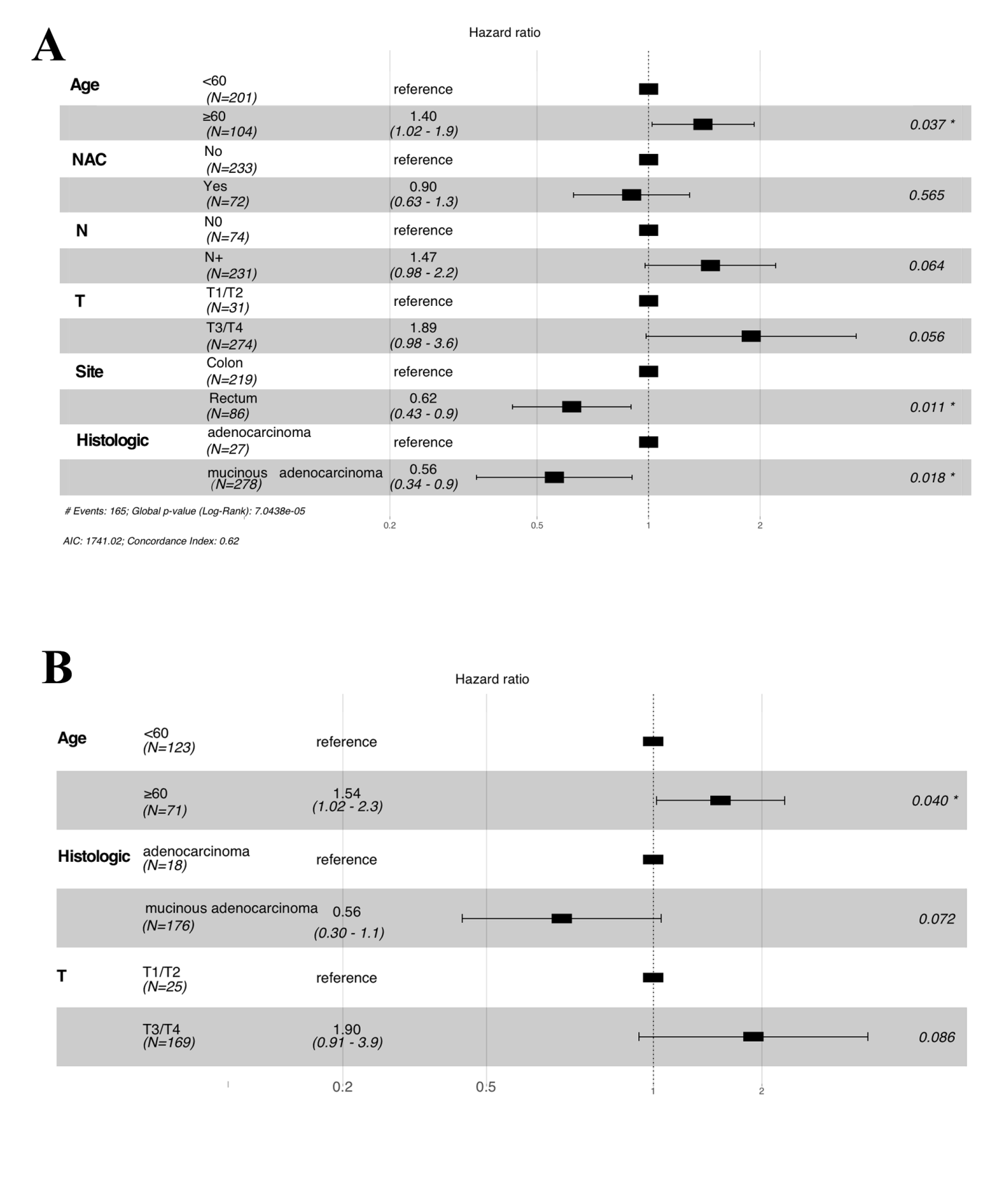


**Supplementary Figure 1**. Forest plot of overall survival in SEER cohort: in the entire cohort (A); in the propensity score-matched cohort (B)


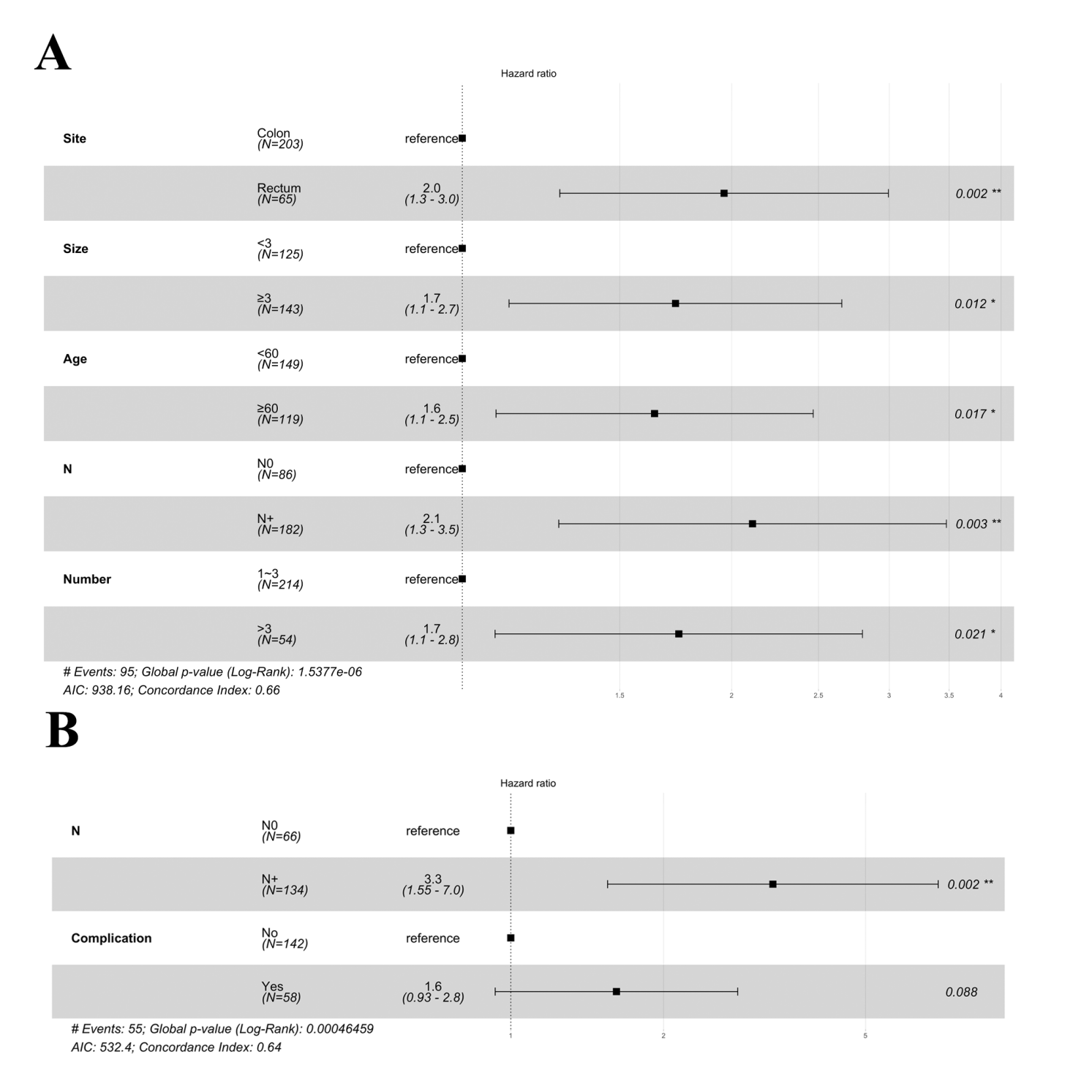


**Supplementary Figure 2**. Forest plot of overall survival in NCC cohort: in the entire cohort (A); in the propensity score-matched cohort (B)


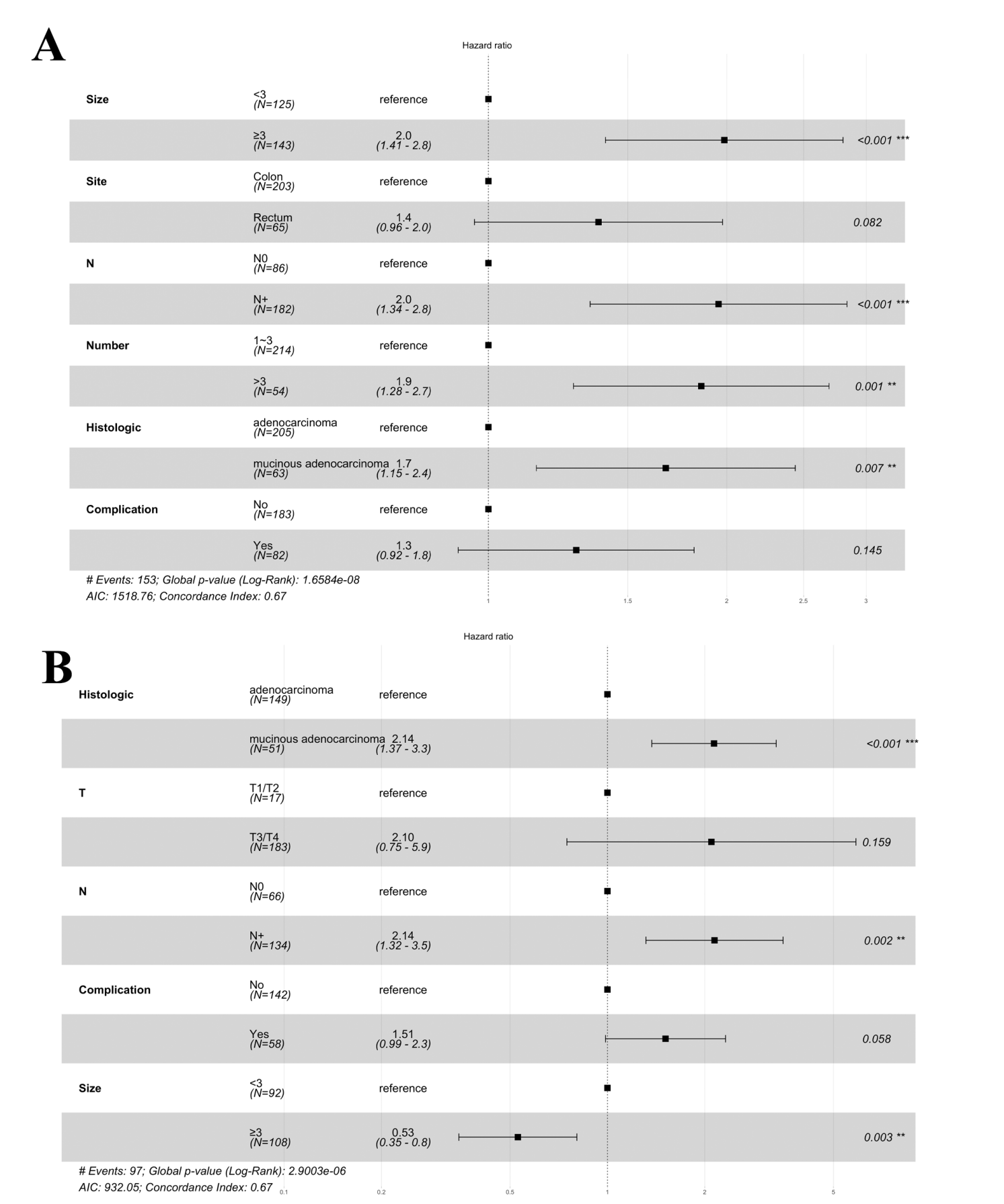


**Supplementary Figure 3**. Forest plot of disease-free survival curves in the NCC cohort: in the entire cohort (A); in the propensity score-matched cohort (B)

**Supplementary Table 1**. Chemotherapy regimen of NAC and AC

| Variable | Before PSM | | After PSM | | |
| --- | --- | --- | --- | --- | --- |
|  | NAC+ (n=88) | NAC- (n=180) | NAC+ (n=76) | NAC- (n=124) | *p* |
| NAC regimen |  |  |  |  |  |
| FOLFOX | 19 | - | 12 | - |  |
| XELOX | 60 | - | 61 | - |  |
| FOLFIRI | 2 | - | 1 | - |  |
| Capecitabine | 3 | - | 2 | - |  |
| Other | 4 | - | 0 | - |  |
| Cycles of NAC |  | - |  | - |  |
| ≤5 | 59 | - | 54 | - |  |
| >5 | 29 | - | 22 | - |  |
| NAC target agent | 23 | - | 22 | - |  |
| AC regimen |  |  |  |  | 0.091 |
| FOLFOX | 12 | 69 | 10 | 30 |  |
| XELOX | 49 | 41 | 43 | 56 |  |
| FOLFIRI | 7 | 27 | 5 | 17 |  |
| Capecitabine | 6 | 10 | 5 | 8 |  |
| Other | 3 | 7 | 3 | 6 |  |
| None | 11 | 26 | 10 | 7 |  |
| Cycles of AC |  |  |  |  | <0.001 |
| ≤5 | 58 | 41 | 52 | 34 |  |
| >5 | 19 | 113 | 14 | 83 |  |
| AC target agent | 24 | 44 | 22 | 42 | 0.469 |

NAC, neoadjuvant chemotherapy; AC, adjuvant chemotherapy; FOLFIRI, folinic acid, fluorouracil, and irinotecan; FOLFOX, folinic acid, fluorouracil, and oxaliplatin; XELOX, capecitabine, and oxaliplatin

**Supplementary Table 2.** Demographic Characteristics of the Patients in SEER Database and NCC Database

| Characteristics | Before PSM | | | After PSM | | |
| --- | --- | --- | --- | --- | --- | --- |
|  | SEER database  (n = 305) | NCC database  (n =268) | *P-value* | SEER database  (n = 194) | NCC database  (n =200) | *P-value* |
| **Age (years)** |  |  | 0.832 |  |  | 0.891 |
| Mean (SD) | 57.8 (10.5) | 58.0 (9.9) |  | 57.9 (10.5) | 57.7 (9.99) |  |
| Median [Min, Max] | 58.0 [31.0, 82.0] | 60.0 [21.0, 78.0] |  | 55.0 [31.0, 82.0] | 60.0 [21.0, 78.0] |  |
| **Sex (%)** |  |  | 0.656 |  |  | 0.390 |
| Male | 180 (59.0%) | 164 (61.2%) |  | 116 (59.8%) | 129 (64.5%) |  |
| Female | 125 (41.0%) | 104 (38.8%) |  | 78 (40.2%) | 71 (35.5%) |  |
| **Site of primary disease (%)** |  |  | 0.330 |  |  | <0.001 |
| Rectum | 86 (28.2%) | 65 (24.3%) |  | 78 (40.2%) | 29 (14.5%) |  |
| Colon | 219 (71.8%) | 203 (75.7%) |  | 116 (59.8%) | 171 (85.5%) |  |
| **Histologic grade (%)** |  |  | <0.001 |  |  | <0.001 |
| Poor/Mucinous/signet | 278 (91.1%) | 63 (23.5%) |  | 176 (90.7%) | 51 (25.5%) |  |
| Moderate | 27 (8.9%) | 205 (76.5%) |  | 18 (9.3%) | 149 (74.5%) |  |
| **pN stage** **(%)** |  |  | 0.047 |  |  | 0.824 |
| N0 | 74 (24.3%) | 86 (32.1%) |  | 61 (31.4%) | 66 (33.0%) |  |
| N+ | 231 (75.7%) | 182 (67.9%) |  | 133 (68.6%) | 134 (67.0%) |  |
| **pT stage (%)** |  |  | 0.686 |  |  | 0.212 |
| T1/T2 | 31 (10.2%) | 31 (11.6%) |  | 25 (12.9%) | 17 (8.5%) |  |
| T3/T4 | 274 (89.8%) | 237 (88.4%) |  | 169 (87.1%) | 183 (91.5%) |  |
| **Neoadjuvant chemotherapy (%)** |  |  | 0.018 |  |  | 0.615 |
| No | 233 (76.4%) | 180 (67.2%) |  | 126 (64.9%) | 124 (62.0%) |  |
| Yes | 72 (23.6%) | 88 (32.8%) |  | 68 (35.1%) | 76 (38.0%) |  |
| **Adjuvant chemotherapy (%)** |  |  | <0.001 |  |  | 0.643 |
| No | 107 (35.1%) | 37 (13.8%) |  | 77 (39.7%) | 85 (42.5%) |  |
| Yes | 198 (64.9%) | 231 (86.2%) |  | 117 (60.3%) | 115 (57.5%) |  |

PSM, Propensity scoring matching; pT: pathologic T stage; pN, pathologic N stage

**Supplementary Table 3** Surgical outcome of patients in the NCC cohort

| Characteristics | Before PSM | | | After PSM | | |
| --- | --- | --- | --- | --- | --- | --- |
|  | Patients without NAC（n=180） | Patients with NAC（n=88） | *P* | Patients without NAC (n=124) | Patients with NAC  (n=76) | *P* |
| **Type of surgery (%)** |  |  | 0.418 |  |  | 1.000 |
| Laparoscopic surgery | 110 (61.1%) | 59 (67.0%) |  | 81 (65.3%) | 49 (64.5%) |  |
| open surgery | 70 (38.9%) | 29 (33.0%) |  | 43 (34.7%) | 27 (35.5%) |  |
| **Operative time (min)** |  |  | **<0.001** |  |  | **<0.001** |
| Mean (SD) | 279 (115) | 353 (135) |  | 278 (113) | 350 (140) |  |
| Median [Min, Max] | 270 [80.0, 660] | 342 [76.0, 800] |  | 280 [90.0, 660] | 339 [76.0, 800] |  |
| **Exhaust time (days)** |  |  | 0.746 |  |  | 0.294 |
| Mean (SD) | 4.27 (1.65) | 4.35 (2.01) |  | 3.95 (1.50) | 4.21 (1.79) |  |
| Median [Min, Max] | 4.00 [2.00, 10.0] | 3.50 [1.00, 12.0] |  | 3.00 [2.00, 9.00] | 3.00 [1.00, 9.00] |  |
| **Hospital stay (days)** |  |  | 0.135 |  |  | 0.680 |
| Mean (SD) | 14.2 (5.75) | 13.0 (6.41) |  | 13.1 (5.35) | 12.8 (5.88) |  |
| Median [Min, Max] | 14.0 [3.00, 28.0] | 11.0 [5.00, 38.0] |  | 13.0 [3.00, 28.0] | 11.0 [5.00, 31.0] |  |
| **Blood loss (ml)** |  |  | 0.286 |  |  | 0.056 |
| Mean (SD) | 240 (349) | 205 (181) |  | 260 (388) | 185 (162) |  |
| Median [Min, Max] | 100 [10.0, 3000] | 200 [20.0, 800] |  | 100 [20.0, 3000] | 200 [20.0, 800] |  |
| **Surgical margin (%)** |  |  | 0.697 |  |  | 1.000 |
| R0 | 158 (87.8%) | 75 (85.2%) |  | 103 (83.1%) | 63 (82.9%) |  |
| R1 | 22 (12.2%) | 13 (14.8%) |  | 21 (16.9%) | 13 (17.1%) |  |
| **Clavien–Dindo Grade (%)** |  |  | 0.114 |  |  | 0.186 |
| Absent | 129 (71.7%) | 58 (65.9%) |  | 94 (75.8%) | 49 (64.5%) |  |
| Ⅰ~Ⅱ | 22 (12.2%) | 7 (8.0%) |  | 10 (8.1%) | 7 (9.2%) |  |
| Ⅲ~Ⅳ | 29 (16.1%) | 23 (26.1%) |  | 20 (16.1%) | 20 (26.3%) |  |

PSM, Propensity scoring matching; NAC, neoadjuvant chemotherapy

**Supplementary Table 4** Prognostic factors for DFS in CRLM patients in the NCC cohort

| Factor | Univariate analyses | |  | Multivariate analyses | |
| --- | --- | --- | --- | --- | --- |
|  | *P-value* | HR (95% CI) |  | *P-value* | HR (95% CI) |
| ***NCC cohort-before PSM*** |  |  |  |  |  |
| Age (≥60y vs <60y) | 0.806 | 0.96 (0.70 - 1.32) |  |  |  |
| Sex (female vs male) | 0.922 | 0.98 (0.71 - 1.36) |  |  |  |
| ASA (Ⅱ vsⅠ) | 0.239 | 1.82 (0.67 - 4.92) |  |  |  |
| (Ⅲ vs Ⅰ) | 0.265 | 1.87 (0.62 - 5.65) |  |  |  |
| pN stage (N+ vs N0) | **0.001** | 1.91 (1.32 - 2.77) |  | **<0.001** | 1.95 (1.34 - 2.84) |
| pT stage (T3+T4 vs T1+T2) | 0.162 | 1.48 (0.85 - 2.56) |  |  |  |
| Histologic grade (Poor/Mucinous/signet vs Moderate) | 0.071 | 1.39 (0.97 - 1.99) |  | **0.007** | 1.68 (1.15 - 2.44) |
| Size of liver metastases (≥3cm vs <3cm) | **<0.001** | 1.84 (1.33 - 2.56) |  | **<0.001** | 1.99 (1.41 - 2.81) |
| Site (Rectum vs Colon) | **0.013** | 1.56 (1.10 - 2.22) |  | 0.082 | 1.38 (0.96 - 1.98) |
| Neoadjuvant Chemotherapy therapy (Yes vs No) | 0.739 | 0.94 (0.65 -1.36) |  |  |  |
| Adjuvant Chemotherapy therapy (Yes vs No) | 0.142 | 0.72 (0.47 - 1.11) |  |  |  |
| Comorbidity (Yes vs No) | 0.175 | 0.80 (0.58 - 1.11) |  |  |  |
| Complication (Yes vs No) | **0.052** | 1.39 (1.00 - 1.94) |  | 0.145 | 1.29 (0.92 - 1.82) |
| Number of liver metastases (≥3 vs <3) | **0.006** | 1.66 (1.15- 2.39) |  | **0.001** | 1.86 (1.28 - 2.69) |
| ***NCC cohort-after PSM*** |  |  |  |  |  |
| Age (≥60y vs <60y) | 0.645 | 0.91 (0.61 - 1.36) |  |  |  |
| Sex (female vs male) | 0.399 | 1.20 (0.79 - 1.83) |  |  |  |
| ASA (Ⅱ vsⅠ) | 0.166 | 2.69 (0.66 - 10.96) |  |  |  |
| (Ⅲ vs Ⅰ) | 0.206 | 2.69 (0.58 - 12.45) |  |  |  |
| pN stage (N+ vs N0) | **0.003** | 2.07 (1.29 - 3.34) |  | **0.002** | 2.14 (1.32 - 3.49) |
| pT stage (T3+T4 vs T1+T2) | 0.055 | 2.67 (0.98 - 7.28) |  | 0.159 | 2.10 (0.75 - 5.87) |
| Histologic grade (Poor/Mucinous/signet vs Moderate) | **0.027** | 1.62 (1.06 - 2.48) |  | **<0.001** | 2.14 (1.37 - 3.33) |
| Size of liver metastases (≥3cm vs <3cm) | **0.007** | 1.73 (1.16 - 2.59) |  | **0.003** | 0.53 (0.35 - 0.80) |
| Site (Rectum vs Colon) | 0.415 | 0.77 (0.41 - 1.44) |  |  |  |
| Neoadjuvant Chemotherapy therapy (Yes vs No) | 0.798 | 0.95 (0.62 -1.44) |  |  |  |
| Adjuvant Chemotherapy therapy (Yes vs No) | 0.710 | 1.08 (0.72 - 1.62) |  |  |  |
| Comorbidity (Yes vs No) | 0.297 | 0.81 (0.54 - 1.21) |  |  |  |
| Complication (Yes vs No) | **0.041** | 1.55 (1.02 - 2.36) |  | 0.058 | 1.51 (0.99 - 2.32) |
| Number of liver metastases (≥3 vs <3) | 0.173 | 1.39 (0.86 - 2.34) |  |  |  |

PSM, Propensity scoring matching; pT: pathologic T stage; pN, pathologic N stage

**Supplementary Table 5.**  Comparison of baseline clinical characteristics based on the number of liver metastases of the NCC cohort

| Characteristics | Patients with<3 metastases  (N=169) | Patients with ≥ 3 metastases  (N=99) | *P-value* |
| --- | --- | --- | --- |
| **Age (years)** |  |  | 0.313 |
| Mean (SD) | 58.5 (10.1) | 57.3 (9.73) |  |
| Median [Min, Max] | 60.0 [21.0, 78.0] | 58.0 [36.0, 77.0] |  |
| **Sex (%)** |  |  | 1 |
| Male | 103 (60.9%) | 61 (61.6%) |  |
| Female | 66 (39.1%) | 38 (38.4%) |  |
| **ASA scores (%)** |  |  | 0.075 |
| Ⅰ | 6 (3.6%) | 4 (4.0%) |  |
| Ⅱ | 142 (84.0%) | 91 (91.9%) |  |
| Ⅲ | 21 (12.4%) | 4 (4.0%) |  |
| **Size of liver metastases (%)** |  |  | 0.399 |
| <3cm | 94 (55.6%) | 49 (49.5%) |  |
| ≥3cm | 75 (44.4%) | 50 (50.5%) |  |
| **Comorbidity (%)** |  |  | 0.365 |
| Absent | 88 (52.1%) | 58 (58.6%) |  |
| Present | 81 (47.9%) | 41 (41.4%) |  |
| **Site of primary disease (%)** |  |  | 0.885 |
| Rectum | 40 (23.7%) | 25 (25.3%) |  |
| Colon | 129 (76.3%) | 74 (74.7%) |  |
| **Histologic grade (%)** |  |  | 1 |
| Poor/Mucinous/signet | 40 (23.7%) | 23 (23.2%) |  |
| Moderate | 129 (76.3%) | 76 (76.8%) |  |
| **pN stage (%)** |  |  | 0.539 |
| N0 | 57 (33.7%) | 29 (29.3%) |  |
| N+ | 112 (66.3%) | 70 (70.7%) |  |
| **pT stage (%)** |  |  | 0.985 |
| T1/T2 | 19 (11.2%) | 12 (12.1%) |  |
| T3/T4 | 150 (88.8%) | 87 (87.9%) |  |
| **Adjuvant chemotherapy (%)** |  |  | 0.126 |
| No | 28 (16.6%) | 9 (9.1%) |  |
| Yes | 141 (83.4%) | 90 (90.9%) |  |
| **Neoadjuvant chemotherapy (%)** |  |  | 0.282 |
| No | 118 (69.8%) | 62 (62.6%) |  |
| Yes | 51 (30.2%) | 37 (37.4%) |  |

pT: pathologic T stage; pN, pathologic N stage
